# Hijacking Factor H for Complement Immune Evasion

**DOI:** 10.3389/fimmu.2021.602277

**Published:** 2021-02-25

**Authors:** Sara R. Moore, Smrithi S. Menon, Claudio Cortes, Viviana P. Ferreira

**Affiliations:** ^1^Department of Medical Microbiology and Immunology, University of Toledo College of Medicine and Life Sciences, Toledo, OH, United States; ^2^Department of Foundational Medical Sciences, Oakland University William Beaumont School of Medicine, Rochester, MI, United States

**Keywords:** complement system, alternative pathway, Factor H, Factor H binding proteins, complement evasion, pathogen

## Abstract

The complement system is an essential player in innate and adaptive immunity. It consists of three pathways (alternative, classical, and lectin) that initiate either spontaneously (alternative) or in response to danger (all pathways). Complement leads to numerous outcomes detrimental to invaders, including direct killing by formation of the pore-forming membrane attack complex, recruitment of immune cells to sites of invasion, facilitation of phagocytosis, and enhancement of cellular immune responses. Pathogens must overcome the complement system to survive in the host. A common strategy used by pathogens to evade complement is hijacking host complement regulators. Complement regulators prevent attack of host cells and include a collection of membrane-bound and fluid phase proteins. Factor H (FH), a fluid phase complement regulatory protein, controls the alternative pathway (AP) both in the fluid phase of the human body and on cell surfaces. In order to prevent complement activation and amplification on host cells and tissues, FH recognizes host cell-specific polyanionic markers in combination with complement C3 fragments. FH suppresses AP complement-mediated attack by accelerating decay of convertases and by helping to inactivate C3 fragments on host cells. Pathogens, most of which do not have polyanionic markers, are not recognized by FH. Numerous pathogens, including certain bacteria, viruses, protozoa, helminths, and fungi, can recruit FH to protect themselves against host-mediated complement attack, using either specific receptors and/or molecular mimicry to appear more like a host cell. This review will explore pathogen complement evasion mechanisms involving FH recruitment with an emphasis on: (a) characterizing the structural properties and expression patterns of pathogen FH binding proteins, as well as other strategies used by pathogens to capture FH; (b) classifying domains of FH important in pathogen interaction; and (c) discussing existing and potential treatment strategies that target FH interactions with pathogens. Overall, many pathogens use FH to avoid complement attack and appreciating the commonalities across these diverse microorganisms deepens the understanding of complement in microbiology.

## The Complement System

**Figure 1:**
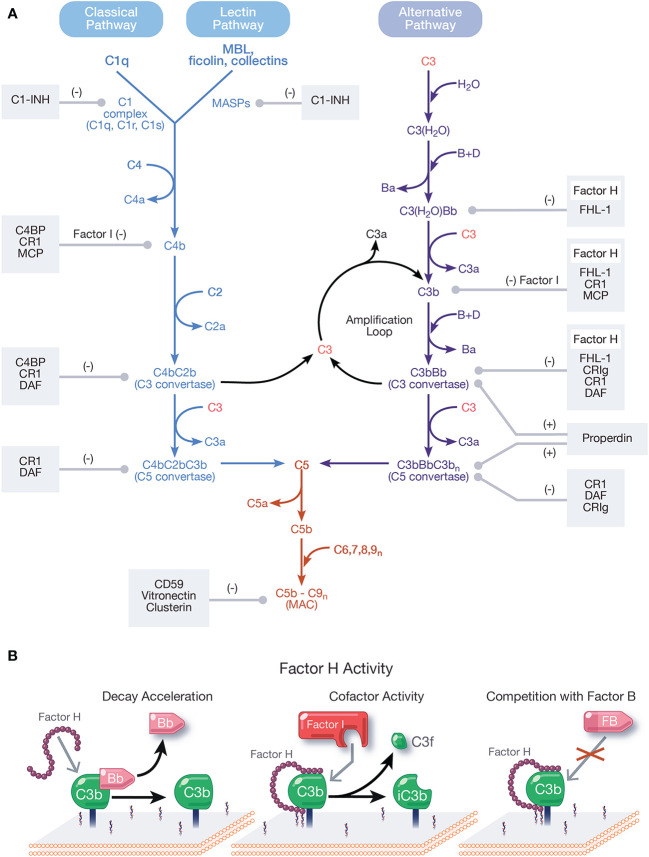
Overview and regulation of the complement system. **(A)** Complement is activated by three pathways: the classical, lectin, and alternative pathways. The classical pathway (CP) activates when the C1 complex (C1q, C1r, C1s) recognizes and binds pathogen- or cell-bound immunoglobulins, circulating immune complexes, or pentraxins. The lectin pathway (LP) activates when mannose-binding lectin (MBL) ficolins, or collectins recognize molecular patterns such as carbohydrates and other ligands on foreign surfaces. CP and LP activation results in cleavage of C4, followed by cleavage of C2, forming the CP/LP surface bound C3 convertase, C4bC2b. The alternative pathway (AP) is spontaneously activated when soluble C3 hydrolyzes to C3(H_2_O). C3(H_2_O) can bind FB (labeled B) and recruit Factor D (labeled D) which cleaves FB to Bb (and Ba), resulting in the fluid phase AP C3 convertase, C3(H_2_O)Bb. C3(H_2_O)Bb cleaves C3 to C3a and C3b. C3b then binds covalently to nearby surfaces to form membrane-bound C3 convertase, C3bBb. C3 convertases derived from all pathways cleave C3 to C3a and C3b. C3b combines with formed C3 convertases to form the CP/LP C5 convertase (C4bC2bC3b) and AP C5 convertase (C3bBbC3b_n_). C5 is cleaved by C5 convertases to initiate the common, terminal pathway, which culminates in the formation of the membrane attack complex (MAC). C3b produced from the cleavage of C3 by C3 convertases from all pathways forms an amplification loop that contributes to the generation of additional AP C3 convertases. Positive (labeled +) and negative (labeled −) regulators of all complement pathways are shown. Membrane-bound complement negative regulators include complement receptor 1 (CR1/CD35), decay accelerating factor (DAF/CD55), membrane cofactor protein (MCP/CD46), CD59, and complement receptor of the immunoglobulin family (CRIg). Soluble negative regulators include Factor H (FH), FI, C4 binding protein (C4BP), C1 inhibitor (C1-INH), clusterin, vitronectin, and Factor-H like protein 1 (FHL-1). Positive regulators of complement include FH-related proteins (FHRs) -1, -4, and -5, and properdin. **(B)** Function of FH. FH accelerates the decay of AP C3 convertase by dissociating Bb (decay acceleration function); acts as a cofactor for Factor I-mediated cleavage of C3b into iC3b, an inactivated form that does not allow complement activity to progress (cofactor activity function); and competes with FB for binding to C3b to form the AP C3 convertase.

Complement activates through a domino-like cascade comprising over 50 proteins, resulting in outcomes essential for innate and adaptive immunity. Activation of complement occurs through three pathways: classical, lectin, and alternative (**Figure 1A**), which converge on the cleavage of the central component, C3 [reviewed in (1)]. The classical pathway (CP) activates when C1q of the C1 complex (C1q, C1r, C1s) recognizes and binds pathogen- or cell-bound immunoglobulins, circulating immune complexes, or to pentraxins (e.g., C-reactive protein, pentraxin 3, serum amyloid P). When C1q binds a ligand, C1r is activated, which then activates C1s. C1s sequentially cleaves C4 and C2, resulting in the CP C3 convertase, C4bC2b [reviewed in (2)]. The lectin pathway (LP) activates when mannose-binding lectin (MBL), ficolins, or collectins recognize molecular patterns such as carbohydrates and other ligands on foreign surfaces. This leads to activation of MBL-associated serine proteases (MASPs), whereby MASP-2 cleaves C4 and C2 to form the LP C3 convertase, C4bC2b [reviewed in (3)].

Unlike the CP and LP, which are triggered upon recognition of distinct ligands, the alternative pathway (AP) is continuously active and initiates spontaneously on surfaces not protected by complement regulatory proteins. In blood, low levels of C3 undergo spontaneous hydrolysis (“tick-over”) to form C3(H_2_O). C3(H_2_O) binds Factor B (FB) and circulating Factor D cleaves FB to Bb and Ba, resulting in formation of the fluid phase AP C3 convertase, C3(H_2_O)Bb. C3(H_2_O)Bb cleaves C3 to C3b and C3b binds covalently to nearby surfaces to form membrane-bound C3 convertase, C3bBb. The AP also contributes to a powerful amplification loop through activation of C3b, which in some cases contributes up to 80% of the total complement response, even after initiation by the CP and LP (4, 5). In fact, it is argued the AP is mainly an amplification mechanism with minimal contributions from the tick-over of C3 [reviewed in (6)].

C3 convertases derived from each pathway converge to cleave C3, generating C3a and C3b, which complexes on or near C3 convertases to form C5 convertases. Cleavage of C5 by C5 convertases generates C5a and C5b to initiate the terminal pathway. Sequential binding to C5b by C6, C7, C8, and multiple copies of C9 form the membrane attack complex (MAC, C5b-9). Outcomes of complement are numerous and include generation of pro-inflammatory mediators C3a and C5a, C3 fragments involved in opsonization and immune modulation, and cell lysis by the pore-forming MAC [reviewed in (7, 8)].

Regulation of all complement pathways protects the host from unwarranted complement-mediated attack. Complement negative regulators circulate in blood and include FH, Factor I (FI), C4 binding protein (C4BP), C1 inhibitor (C1-INH), clusterin, vitronectin, and Factor-H like protein 1 (FHL-1). Membrane-bound complement negative regulators include complement receptor 1 (CR1/CD35), decay accelerating factor (DAF/CD55), membrane cofactor protein (MCP/CD46), CD59, and complement receptor of the immunoglobulin family (CRIg) [reviewed in (9)]. Importantly, FH is the primary regulator of the AP in the fluid phase and on cell surfaces and is essential for protecting the host from AP attack. Pathogens have developed survival strategies to evade the immune response including coopting FH from the host to avoid the AP. This phenomenon will be described in detail in this review.

## Factor H in the Host

FH, formerly known as β1H (10, 11), is abundantly found in plasma with a wide concentration range between 116 and 810 μg/ml [reviewed in (12, 13)]. However, recent studies reveal an average FH concentration of ~230 μg/ml, when measurement of FH family proteins FHL-1 and Factor-H related proteins (FHRs) are excluded [reviewed in (14)]. FH is constitutively expressed by hepatocytes (15, 16) and also produced by monocytes, fibroblasts, endothelial cells, platelets, retinal pigment epithelial cells, peripheral blood lymphocytes, myoblasts, rhabdomyosarcoma cells, glomerular mesangial cells, neurons, and glial cells [reviewed in (12, 17)]. FH is a 155-kDa glycoprotein (18, 19) encoded from a single gene, HF1/CFH, found within the regulator of complement activation gene cluster on chromosome 1q32 [reviewed in (20)]. FH consists of 20 homologous complement control protein modules (CCP) (21, 22), with each module containing ~60 amino acid residues (22) connected by short spaces of three to eight amino acid residues [reviewed in (17)]. Structural studies indicate FH may adopt a flexible folded back conformation in solution (23–25).

FH is the primary negative regulator of the AP in the fluid phase and at the cell surface. The three functions of FH include: (a) competing with FB for C3b binding (26); (b) accelerating the decay of surface-bound C3 and C5 convertases (10, 27, 28) and to a lesser extent, fluid-phase C3 convertase (29), by disassociating Bb from convertases; and (c) acting as a cofactor for FI-mediated cleavage of C3b into the inactive form, iC3b (28, 30, 31) (**Figure 1B**). These regulatory functions are carried out through CCPs 1–4 (32–35).

**Figure 2:**
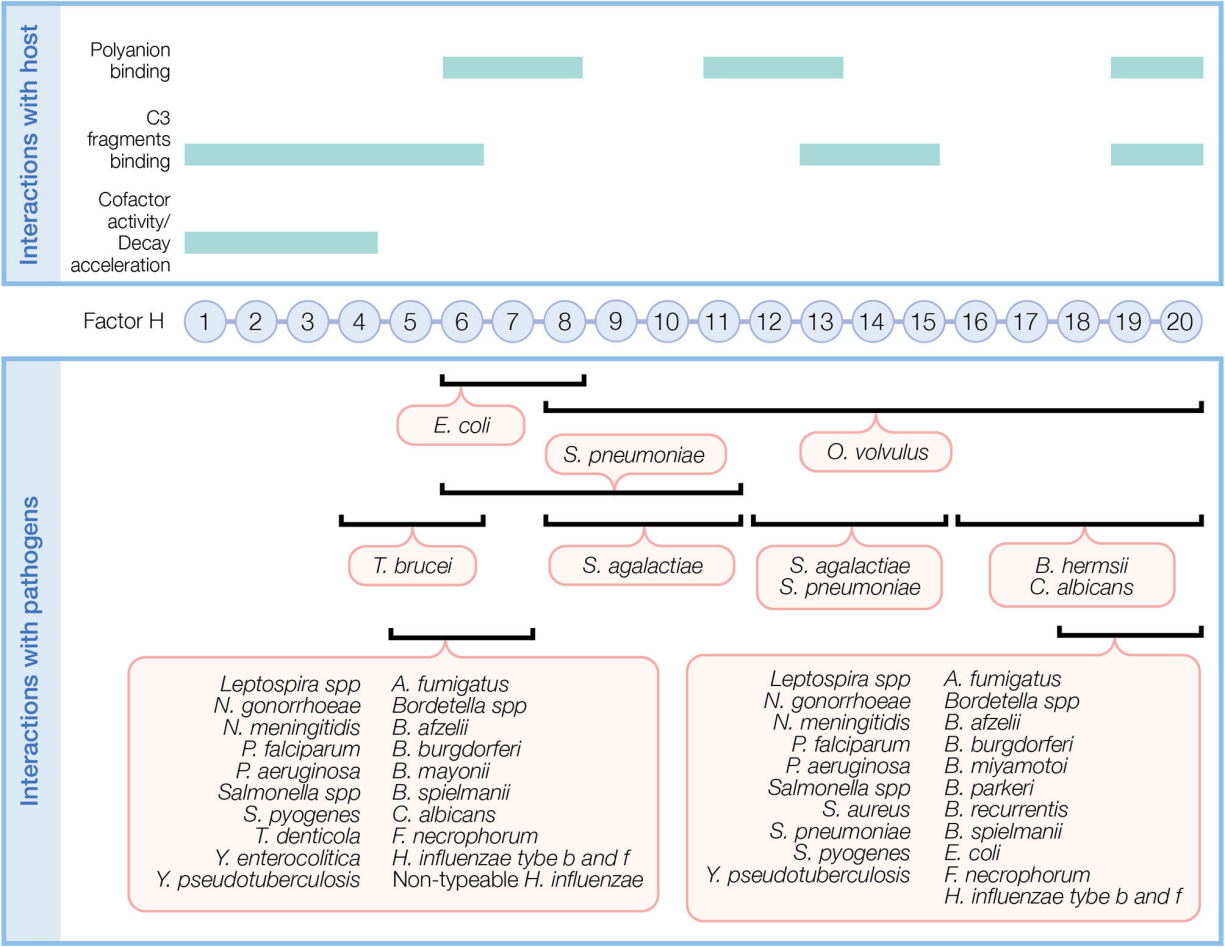
Factor H domains involved in binding to the host and pathogens. FH contains 20 complement control protein modules (CCP) each with about 60 amino acids linked by three to five amino acids. In the top panel, FH domains necessary for its regulatory function and domains interacting with C3 fragments and polyanions are indicated. In the bottom panel, overlapping FH domains involved in binding pathogens are indicated. Refer to **Table 1** for specific FH domains involved in binding to individual pathogens and for pathogens binding FH through undefined domains.

Upon binding to a surface, FH protects against AP activity. FH simultaneously recognizes C3 fragments and host cell markers to discriminate self (host, non-activators) from non-self (activators), which lack or have very low levels of surface polyanions. Several FH domains participate in binding C3 fragments and/or polyanions (**Figure 2**, top panel). CCPs 1–6 bind C3 and C3b and a weak binding site for C3b is recognized at CCPs 13–15 (36). CCPs 19–20 recognize iC3b, C3b, and C3d (36, 37). Host cell polyanions, such as sialic acids (38, 39) and glycosaminoglycans (GAGs), which include heparins (40, 41) and dextran sulfate (41, 42), serve as recognition markers for FH regulation on host cells. CCP 20 is the only known site to recognize sialic acid (43). Heparin binding sites include CCPs 6–8 and 18–20, with a possible weak binding site on CCPs 11–13 (36). CCPs 19–20 are the most important region of FH for binding to cell surfaces by recognizing both C3b and polyanions [reviewed in (44)]. FH has a 10-fold increase in affinity towards C3b in the presence of host sialic acid (38, 39, 41). In addition to recognizing host cell markers, FH acts as a ligand for annexin II, DNA, C-reactive protein, and pentraxin-3 to limit excessive complement activation during apoptosis [reviewed in (44)].

FH belongs to a family of proteins including FHL-1 and five FHRs, reviewed elsewhere (14, 45), that are present at significantly lower concentrations than FH in blood [reviewed in (14)]. Briefly, FHL-1 is a result of alternative splicing of CFH and contains seven domains homologous to FH CCPs 1–7 plus four C-terminal amino acids. FHL-1 is a negative regulator of complement in the fluid phase [reviewed in (14, 45, 46)]. In contrast, FHRs do not share a gene with FH or FHL-1, but instead, have domains homologous to the center and C-terminal FH domains [reviewed in (14)]. Thus, due to a non-functional N-terminus, FHRs compete with FH, thereby acting as potential positive regulators of the AP, yet the roles of FHRs are not well defined [reviewed in (14, 45)].

In addition to the prototypical role of FH as a negative regulator of the AP, non-canonical roles of FH have emerged [reviewed in (45)]. FH is a ligand for complement receptor 3 (CR3; CD11b/CD18) on neutrophils and upon binding, results in release of hydrogen peroxide and lactoferrin (47). FH interaction with neutrophil CR3 also results in release of IL-8, prevention of neutrophil extracellular traps (NETs) formation, and production of reactive oxygen species (ROS) (48). Thus, FH may reduce host damage by inhibition of NETs and ROS (48). In particular, FH bound to *Candida albicans* modulates neutrophil function by interacting primarily with CR3, and to a lesser extent to complement receptor 4 (CR4; CD11c/CD18), leading to more effective killing of the pathogen (49). FH is also known as adrenomedullin-binding protein-1 and binds adrenomedullin (50), a vasodilator peptide hormone widely expressed in many human tissues [reviewed in (51)]. FH may protect adrenomedullin from proteolytic degradation and thus has therapeutic value in disease models of sepsis, wound healing, and hemorrhage [reviewed in (52)]. In addition to acting as a ligand, FH is internalized by early apoptotic cells, resulting in enhancement of intracellular C3 cleavage and increased iC3b surface opsonization to promote uptake by monocytes [reviewed in (45)].

## Factor H Interactions With Pathogens

Evasion of complement attack is key to pathogen survival in the host. In general, pathogens evade complement through numerous strategies, which include: (a) expression of proteins that mimic host surface-bound complement regulators; (b) secretion of proteases to digest complement fragments; (c) exploitation of complement opsonization to promote intracellular invasion; (d) secretion of complement inhibitory proteins; and (e) recruitment of fluid phase complement regulators, which includes FH family proteins [reviewed in (53–55)].

Pathogen evasion of the complement system has been described [reviewed in (54–58)], including strategies particular to bacteria [reviewed in (53, 59)]; fungi [reviewed in (60)]; parasites [reviewed in (61)]; viruses [reviewed in (62)]; and evasion mechanisms involving FH family proteins [reviewed in (17, 19, 63)]. Herein, we review how pathogens steal FH to outsmart the immune system. Specifically, we describe the implications of FH binding in pathogen evasion, mechanisms of binding, and therapeutic targeting of the FH-pathogen interface.

### Binding of Factor H to Pathogens to Control Alternative Pathway Activity

FH is the primary target of pathogens for AP evasion. When sequestered from blood to the pathogen surface, FH retains its function as a negative regulator of complement, thus circumventing lysis by MAC, opsonization by C3 fragments, and pro-inflammatory consequences of complement cleavage products such as C3a and C5a [reviewed in (7, 8)].

### Binding of Factor H to Pathogens for Purposes Other Than Alternative Pathway Evasion

Though FH is primarily a negative regulator of the AP, it has also been shown to regulate the CP [reviewed in (64)]. Moreover, FH binding to certain pathogens facilitates host cellular adherence and invasion. Binding of FH to pneumococcal surface protein C (PspC) on S*treptococcus pneumoniae* increases attachment to, and invasion of, host cells (65). Likewise, in *Mycoplasma hyopneumoniae*, FH binding increases adherence to epithelial cells (66) while FH interaction with influenza A virus promotes viral cellular invasion. As opposed to binding FH to evade complement attack, pathogens also cleave FH. Example of pathogens that cleave and inactivate FH include *Salmonella enterica* (67), *Yersinia pestis* (67), *C. albicans* (68), and *Treponema denticola* (68, 69). Inactivation of FH by pathogen proteases may result in unchecked AP activation, consequentially depleting complement proteins surrounding the pathogens, thus protecting them from attack. In addition, FH inactivation leads to complement dysregulation on host cells, which, as suggested by Riva et al. and Miler et al., may compromise tissue integrity to facilitate pathogen invasion (67, 70).

### Binding of Factor H Family Members to Pathogens

FHL-1 and FHRs are also capable of binding pathogens (**Table 1**). Pathogen recruitment of FHL-1 serves the same purpose in complement evasion as binding to full-length FH. However, as suggested by Kunert et al., low serum FHL-1 titers, along with the increased binding affinity of full-length FH because of its additional C-terminal binding domains, may limit pathogen binding to FHL-1 (132). Interestingly, although most *Borrelia* species bind FH, *B. burgdorferi* Complement Regulator-Acquiring Surface Proteins (CRASP)-2 (Csp-Z) is shown to preferentially bind FHL-1 (183).

**Table 1:** Pathogen interactions with Factor H family proteins.

Pathogen	Factor H binding ligand(other name designations)	Binding domains of Factor H family proteins	References
**Bacteria**			
***Acinetobacter baumannii***	AbOmpA	FH	(71)
***Bacillus anthracis***	BclA	FH	(72)
***Bordetella parapertussis, pertussis***	?	5–7 (FH, FHL-1); 19–20 (FH); FHR-1	(73, 74)
***Borrelia afzelii***	CspA (BaCRASP-1)	5–7 (FH, FHL-1)	(75–78)
	CspZ (CRASP-2, BBH06)	6–7 (FH, FHL-1)	(75, 77, 78)
	BaCRASP-3	6–7 (FHL-1)	(77)
	BaCRASP-4	19–20 (FH)	(77)
	BaCRASP-5	19–20 (FH)	(77)
	BAPKO_0422	FH	(79)
***Borrelia burgdorferi***	CspA (CRASP-1, BbCRASP-1, BBA68, FHBP)	5–7 (FH, FHL-1); 19–20 (FH)	(75, 77, 80)
	CspZ (CRASP-2, BbCRASP-2, BBH06)	7 (FH, FHL-1)	(77, 81)
	ErpP (CRASP-3, BbCRASP-3, BBN38)	19–20 (FH); FHR-1, -2, -5	(77, 82, 83)^#^
	ErpC (CRASP-4, BbCRASP-4)	FH; FHR-1, -2	(77, 83)^#^
	ErpA (CRASP-5, BbCRASP-5, ErpI, ErpN, BBP38, BBl39, OspE)	19–20 (FH); FHR-1, -2, -5	(74, 77, 83–85)^#^
***Borrelia hermsii***	BhCRASP-1 (FhbA, FHBP19)	16–20 (FH); FHL-1; FHR-1	(86–88)
***Borrelia mayonii***	CspA	5–7 (FH, FHL-1)	(89)
***Borrelia miyamotoi***	CbiA	20 (FH)	(90)
***Borrelia parkeri***	BpcA	19–20 (FH); FHR-1	(91)
***Borrelia recurrentis***	HcpA	19–20 (FH); FHR-1	(92)
	?	FH	(93)
***Borrelia spielmanii***	CspA (BsCRASP-1)	5–7 (FH, FHL-1)	(94–96)
	BsCRASP-2	FH; FHL-1	(94)
	BsCRASP-3	20 (FH); FHL-1; FHR-1	(94, 96)
***Escherichia coli***	Stx2	6–8 (FH, FHL-1); 18–20 (FH); 3–5 (FHR)	(97, 98)
	OmpW	FH	(99)
***Francisella tularensis***	?	FH	(100)
***Fusobacterium necrophorum***	?	5–7 (FH, FHL-1); 19–20 (FH); FHR-1, -4	(101)
***Haemophilus influenzae type b and f***	Protein H (PH)	7 (FH, FHL-1); 18–20 (FH)	(102, 103)
***Histophilus somni***	?	FH	(104)
***Leptospira spp***	LenA (LfhA, Lsa24)	18–20 (FH, FHR-1)	(105)
	Len B	FH	(106)
	LcpA	20 (FH)	(107)
	LigA, LigB	5–7 (FH, FHL-1); 18–20 (FH); FHR-1	(108, 109)
	Lsa23	FH	(110, 111)
	LIC11966/ErpY-like lipoprotein	FH	(112)
	EF-Tu	FH	(113)
	?	FH	(114)
	Enolase	FH	(115)
***Moraxella catarrhalis***	OlpA	FH	(116)
***Mycoplasma hyopneumoniae***	EF-Tu	FH	(66)
***Neisseria cinerea***	FHbp	FH	(117)
***Neisseria gonorrhoeae***	PorB.1A (PorB1a, Por1A)	6 (FH, FHL-1); 18–20 (FH); FHR-1	(118, 119)
	PorB.1B (PorB1b, Por1B)	18–20 (FH)	(118–120)
	NspA	6–7 (FH, FHL-1)	(121)
***Neisseria meningitidis***	FHbp	6–7 (FH)	(122, 123)
	NspA	6–7 (FH, FHL-1)	(124)
	PorB2	6–7 (FH, FHL-1)	(125)
	PorB3	6–7 (FH)	(126)
	LOS sialylation	18–20 (FH)	(127)
**Non‐typeable *Haemophilus influenzae***	OmpP5 (P5)	6–7 (FH)	(128, 129)
***Pasteurella pneumotropica***	?	FH	(130)
***Pseudomonas aeruginosa***	Lpd	7 (FH, FHL-1); 18–20 (FH); 3–5 (FHR-1)	(131)
	Tuf	6–7 (FH, FHL-1); 19–20 (FH); 3–5 (FHR-1)	(74, 132)
***Rickettsia conorii***	OmpB β-peptide	FH	(133)
***Salmonella spp***	Rck	5–7, 19–20 (FH)	(134)
***Staphylococcus aureus***	SdrE	20 (FH)	(135, 136)
	Sbi	19–20 (FH); FHR-1	(137)
***Streptococcus agalactiae***	β protein (Bac, β C)	8–11, 12–14 (FH)	(138, 139)
	Sht I and II	FH	(140, 141)
***Streptococcus pneumoniae***	PspC (CbpA, SpA, Hic, C3-binding protein)	6–10, 8–10, 9, 8–11, 19–20, 13–15 (FH)	(65, 139, 142–148)
	Tuf	6–7 (FH, FHL-1); 18–20 (FH); 3–5 (FHR-1)	(149)
	LytA	FH	(150)
***Streptococcus pyogenes***	Fba	7 (FH, FHL-1)	(151, 152)
	M protein family	7 (FH, FHL-1)	(153–155)
	Scl1	19–20 (FH); 3–5 (FHR-1)	(156, 157)
***Streptococcus suis***	Fhb	FH	(158)
	Enolase, EF-Tu, PK, GAPDH, FBA, FBPS, KAR, MRP1	FH	(99)
***Treponema denticola***	FhbB	7 (FH)	(68, 70, 159)
***Yersinia enterocolitica***	YadA	FH	(160)
	Ail	6–7 (FH)	(160)
***Yersinia pseudotuberculosis***	Ail	5–7, 19–20 (FH)	(161)
**Fungi**			
***Aspergillus fumigatus***	AfEno1	6–7 (FH, FHL-1); 19–20 (FH)	(162)
	Aspf2	6–7 (FH, FHL-1); 19–20 (FH); 3–5 (FHR-1)	(163)
***Candida albicans***	Pra1	5–7 (FH, FHL-1); 16–20 (FH)	(164)
	Gpm1p (CRASP 1)	6–7 (FH, FHL-1); 19–20 (FH)	(165)
	Gpd2 (Gapdh)	7 (FH, FHL-1)	(166)
	Hgt1p	6–7 (FH)	(167, 168)
**Protozoa**			
***Trypanosoma brucei***	FHR	4–6 (FH)	(169)
***Trypanosoma cruzi***	?	FH	(170)
***Plasmodium falciparum***	?	5 (FH, FHL-1); 20 (FH); FHR-1	(171)
	PfGAP50	5–7 (FH, FHL-1)	(172)
	Pf92	5–6 (FH, FHL-1)	(173)
**Helminths**			
***Toxoplasma gondii***	?	FH	(174)
***Onchocerca volvulus***	?	(8–20) FH	(175)
***Echinococcus granulosus***	?	FH	(176)
***Loa Loa***	?	FH	(177)
**Viruses**			
***West Nile virus***	NS1	FH	(178)
***Human immunodeficiency virus-1***	gp41, gp120	FH	(179–182)

^#^Reviewed in.

The benefits of pathogens binding FHRs are shown when Scl of *S. pyogenes* binds FHR-1 to inhibit the formation of the terminal complement pathway (157). However, to date, most studies suggest pathogen binding to FHRs are disadvantageous, because it outcompetes full-length FH for binding to pathogen surfaces and can enhance AP activity [reviewed in (45)]. In *S. pyogenes* (157) and *P. falciparum* (184), FHR-1 outcompetes FH for binding and impairs FH cofactor activity. Moreover, in the case of *P. falciparum*, FHR-1 binding also leads to decreased parasite viability (184). In *N. meningitidis*, FHR-3 outcompetes FH binding to result in complement-mediated bacteria lysis (185). *Borrelia* CRASP-3 (ErpP) and -5 (ErpA) bind FHR-1, -2, and -5 while only weakly binding FH (186). However, since FHRs binding does not provide any complement evasion benefits to *Borrelia* (186), the consequences of FHRs binding are unclear.

## Mechanisms of How Pathogens Bind Factor H to Evade the Alternative Pathway

Pathogens capture FH by adapting their surfaces to mimic host cells (i.e., expressing host cell markers) and/or expressing specific FH binding receptors (FH binding proteins). Pathogens must bind FH using accessible domains that do not interfere with the N-terminal functions of FH. The sections below describe mechanisms used by pathogens to bind FH. **Figure 2** (bottom panel) illustrates regions of FH recognized by pathogens. Moreover, **Table 1** summarizes pathogens known to bind FH, FHL-1, and FHRs.

### The Role of Sialylation in Factor H Recruitment

Pathogens evade complement by protecting their surfaces from complement attack using host cell markers. In the host, FH recognizes specific sialic acid capped glycans on host surfaces to distinguish self from non-self, which importantly restricts AP activation on self surfaces, while allowing AP activity to continue on non-self (pathogen) surfaces. However, pathogens bypass AP attack by expressing host glycans (187) which go on to capture FH.

α-N-acetylneuraminic acid (Neu5Ac) is a sialic acid species present on pathogens and humans. FH exclusively binds Neu5Ac species with α(2,3) linkages (43), discriminating against pathogens expressing other Neu5Ac linkages including α(2,6) and α(2,8) [reviewed in (188, 189)]. *Neisseria meningitidis* and *Neisseria gonorrhoeae* are Gram-negative bacteria restricted to humans that express α(2,3) linked Neu5Ac which participate in FH binding and other complement evasion tactics [reviewed in (188–190)].

**Figure 3:**
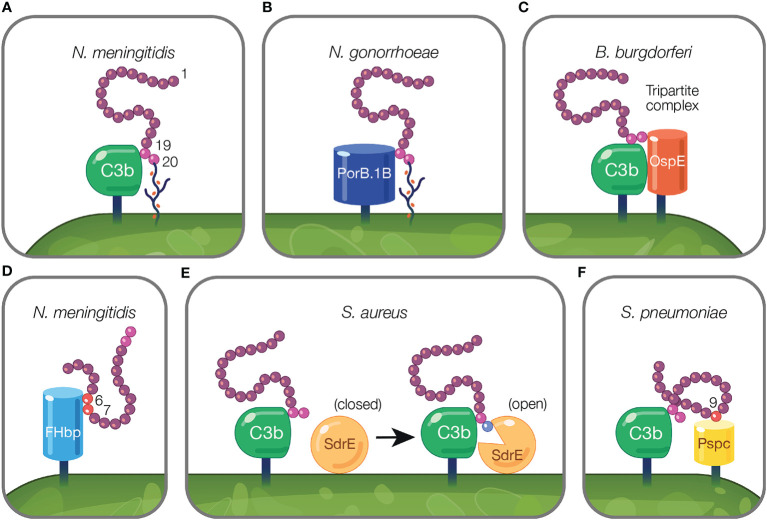
Mechanisms of pathogens binding Factor H to evade the alternative pathway. **(A)** Pathogens evade complement by protecting their surfaces from complement attack using host cell markers. Neu5Ac sialylation of *N. meningitidis* replaces host GAGs and binds FH CCP 20 domain while maintaining the interaction between CCP 19 and deposited C3 fragments (127). **(B)** Pathogen sialylation promotes FH binding in the absence of complement fragments. *N. gonorrhoeae* FH binding protein, PorB.1B, binds FH CCP 19 domain replacing the interaction of C3b with FH CCP 19 [reviewed in (189)]. **(C, D)** Pathogen FH binding proteins mimic interactions between host and FH. **(C)** *B. burgdorferi* FH binding protein, OspE, forms a tripartite complex with FH where CCP 19 binds to C3 fragments and CCP 20 interacts with OspE. **(D)** *N. meningitidis* binds FH through its FH binding protein, FHbp, *via* FH CCPs 6–7. **(E, F)** Pathogen FH interactions that do not mimic mechanisms utilized by the host. **(E)** FH binding to deposited C3b on the pathogen surface results in a transformational change in *S. aureus* FH binding protein, SdrE, from a closed to open state. In the open state, SdrE facilitates the docking of CCP 20 into a ligand binding groove (136). **(F)** *S. pneumoniae* FH binding protein, PspC, *via* its tyrosine 90 residue acts like a key inserting into a hydrophobic lock formed by four hydrophobic residues of FH CCP 9 (146).

On the activated host surface, FH domain CCP 20 interacts with GAGs and CCP 19 binds C3b (191). Sialylated *N. meningitidis* likely recreates this interaction on the pathogen surface in order to bind FH. To bind FH, *N. meningitidis* and *N. gonorrhoeae* sialylate lacto-N-neotetraose (LNnT) branches of lipooligosaccharides (LOS) with Neu5Ac [reviewed in (188)]. Neu5Ac sialylation of *N. meningitidis* LOS enhances FH binding to pathogen surfaces (127) (**Figure 3A**). This is postulated to occur when sialylation on the pathogen surface replaces host GAGs as the ligand for CCP 20, while maintaining the interaction between CCP 19 and deposited C3 fragments [reviewed in (188)]. Similarly, binding of bovine FH to *Histophilus somni* increases when bacteria are sialylated with Neu5Ac (104). While pathogen sialylation promotes FH binding to the cell surface, it still renders pathogens vulnerable to complement-mediated damage because a portion of the deposited C3 fragments involved in binding FH will likely form C3 convertases as opposed to binding FH [reviewed in (190)].

Pathogen sialylation also promotes FH binding in the absence of complement fragments. Increased binding of FH to *N. gonorrhoeae* occurs with sialylation of LNnT LOS (120, 192) only with the concomitant presence of gonococcal FH binding protein, porin B.1B (PorB.1B) (192) (**Figure 3B**). This phenomenon is speculated to occur through an interaction similar to what is described for *N. meningitidis*, but with PorB.1B replacing the interaction of C3b with FH CCP 19 [reviewed in (189)], thus eliminating the need for deposited C3 fragments.

Sialylation also increases binding of FH CCPs 6-7 to Neisserial surface protein A (NspA) of *N. meningitidis* (124). Here, bacterial sialic acid potentially acts as a docking station for FH, perhaps through binding CCP 20, but this has yet to be shown experimentally and may be structurally impossible, as suggested by Lewis et al. (124). In contrast, in *N. gonorrhoeae*, LOS sialylation impedes binding of FH CCPs 6-7 to NspA (121).

α-N-glycolylneuraminic acid (Neu5Gc) is a sialic acid variant not expressed by humans, but present in mice (193) and on the surface of sheep erythrocytes (194). Human FH binds Neu5Gc in a similar manner as Neu5Ac (195) and *N. gonorrhoeae* LNnT-LOS incorporation of Neu5Gc results in FH binding and serum resistance (196). As noted by Schmidt et al., this phenomenon may skew conclusions from animal research, including infection models using humanized FH transgenic mice, as FH binding may be affected in a manner not possible in humans (195).

### Pathogen Factor H Binding Proteins

In addition to decorating their surfaces with host markers, certain pathogens capture FH directly using binding proteins alone. While many FH binding proteins have been identified along with the regions to which they bind on FH, the mechanisms explaining many of these interactions remain unresolved. Below, we describe two strategies employed by pathogen FH binding proteins to capture FH. In the first mechanism, FH binding proteins mimic binding interactions present in the host, but using protein-protein interactions instead of binding to polyanions on host surfaces. In these instances, FH binding proteins bind to the same domains on FH (CCPs 6–7 and 19–20) that are used to bind to the host. In the second mechanism, FH binding proteins capture FH *via* domains (i.e., amino acid residues) on FH that do not bind to the host.

#### Pathogen Factor H Binding Proteins Mimic Interactions Between Host and Factor H

In this section, we describe how FH binding proteins capture FH by binding to the regions on FH that would normally bind to the host. However, unlike host surfaces, which bind FH through expression of host cell markers (i.e., polyanions) in combination with C3 fragment, the binding of FH to the FH binding protein on the pathogen, constitutes solely a protein-protein interaction instead.

An excellent example of FH interactions with a FH binding protein was demonstrated for the FH binding protein of *N. meningitidis*, FHbp (**Figure 3D**). Crystallography studies revealed FHbp binds FH through extensive interactions between β-barrels of FHbp and CCP 6, and through minor contacts with CCP 7 (122). The site within CCP 6 utilized by FHbp (122) overlaps with a previously described binding location for sucrose octasulphate (SOS), a highly sulphated analog of GAGs (197). Similarly, the binding domain for FhbB from *T. denticola* also overlaps with the SOS binding domain (70). Together, these examples demonstrate how FH binding proteins mimic interactions between FH and host charged sugars through amino acid side chains (122).

Protein motif mimicry also involve pathogen interactions with FH C-terminal domains. FH binds host surfaces through dual recognition when CCP 20 binds GAGs and CCP 19 binds the C3d part of C3b (191). Similarly, in the process of mimicking host surfaces, FH binding proteins form a tripartite complex when FH CCP 19 binds C3 fragments and CCP 20 interacts with a FH binding protein (**Figure 3C**). This phenomena has been described for FH binding proteins of *Pseudomonas aeruginosa* (Tuf) (74), *Borrelia burgdorferi* (OspE) (74, 85, 198), *Borrelia hermsii* (FhBA) (74), and *Staphylococcus aureus* (Sbi*)* (137).

In *B. burgdorferi*, crystallography studies indicate OspE (a paralog of CRASP-3) forms a tripartite complex between FH and C3 fragments. This occurs when loops β2–4 and the interface between loops β5–6 interact with CCP 20 which includes FH amino acids overlapping with those involved in heparin binding, while deposited C3dg interacts with CCP 19 (85, 198). The majority of amino acid residues utilized by OspE for binding FH are conserved across the OspE protein family (85). Interestingly, ectopic expression of *B. burgdorferi* CRASP-3 in a serum sensitive *Borrelia* strain bound minute amounts of FH and did not confer serum protection (186). As suggested by Kolodziejczyk et al., the FH tripartite complex may not form when OspE is ectopically expressed, leading to inadequate complement protection (198). Alternatively, according to Siegel et al., additional complement binding proteins may be required to unfold FH to a conformation sufficient for interacting with binding proteins, which does not occur when ectopically expressed (186). While this study examined tripartite complex formation under conditions in which the complex is not surface-bound, this formation is possible under physiological conditions because the domains responsible for tethering OspE to the surface do not interfere with the binding site for FH and bound FH is orientated toward the surface to which C3b is bound *via* the thioester bond (85).

#### Pathogen Factor H Binding Proteins Sequester Factor H Using Interactions Not Found in the Host

Pathogen interactions with FH have been described that do not reflect mechanisms utilized by the host. In the model proposed by Zhang and colleagues, *S. aureus* surface protein serine–aspartate repeat protein E (SdrE) tightly binds a 21 amino acid region of CCP 20 not involved in the binding of C3d or of host cell markers (i.e., heparin, GAGs, and sialic acid) (85, 136). Upon FH binding to deposited C3b on the pathogen surface, SdrE undergoes a transformational change from a close to open state, which facilitates the docking of CCP 20 into a ligand binding groove (136). SdrE functions as a “clamp” to stabilize the SdrE-FH complex by locking and latching the FH tail into its ligand binding groove (136). The “close, dock, lock, and latch” mechanism is similar to strategies utilized by members of the microbial surface components recognizing adhesive matrix molecules, to which SdrE belongs (136). The described mechanism of SdrE is unique in the field of complement immune evasion [reviewed in (199)] (**Figure 3E**).

*S. pneumoniae* FH binding protein, PspC, also binds FH through a domain less utilized by other pathogens. PspC binds CCP 9 when the tyrosine 90 residue of PspC inserts like a key into a hydrophobic lock formed by four hydrophobic residues of FH (146) (**Figure 3F**). Studies using PspC lacking the residue important for interacting with the hydrophobic lock, showed PspC forms stable complexes with FH CCPs 8–10 (145). Here, PspC induces a functionally enhanced FH conformation that accelerates C3bBb decay 5-fold compared to FH alone, and increases the ability of FH to bind C3b 2-fold (145). Herbert et al. suggest a similar process may occur in the host in which host cell markers on host surfaces bind FH, and result in configurations of FH with enhanced regulatory activity.

## Role of Factor H Binding Proteins in Pathogen Virulence

The necessity of FH binding proteins in pathogen virulence is questionable. While most of the described interactions between pathogens and FH demonstrate the advantage of binding FH in AP evasion through *in vitro* assays, *in vivo* studies are essential for further characterizing the role of FH binding in pathogen virulence. The importance of FH in pathogen virulence varies according to pathogen. For example, inhibition of FH binding is protective in several pathogens as supported by studies in which pathogen FH binding is blocked with recombinant FH proteins, or through vaccination with FH binding proteins (discussed in *Therapeutic and Preventive Strategies Targeting the Pathogen-Factor H Interface*). In addition, *in vivo* studies involving the FH binding protein, plasmodial transmembrane protein gliding associated protein 50 (PfGAP50) of *Plasmodium falciparum* was shown to be important in pathogen virulence when antibody neutralization of PfGAP50 reduced parasite transmission to the mosquito vector (172).

*In vitro* serum sensitivity of *Borrelia* species is associated with FH binding potential, considering almost all serum-resistant species recruit FH [reviewed in (200)]. However, *in vivo* studies demonstrate FH binding to pathogens is not required for infection. Numerous studies in *Borrelia* indicate FH may be dispensable for infection. For instance, FH-deficient mice, and mice without FB or C3 (no AP activity), were infected with *Borrelia* at levels similar to wild type animals (201). This finding suggests additional complement-dependent and -independent immune evasion strategies of *Borrelia* replace the benefits of FH capture for survival in the host (201). *B. garinii* is responsible for causing Lyme disease, but does not bind FH, highlighting that FH may be dispensable for infection (202). The mechanisms by which *B. garinii* evades complement to establish infection in the host remain unknown [reviewed in (200)]. Similarly, CspA (CRASP-1) variants are not required for spirochete survival at the mouse tick bite site, though it is expressed at this location (203). Additionally, FH binding protein, FhbA of *B. hermsii* is not required for murine infection or human serum resistance (204). *B. hermsii* does not express additional FH binding proteins, suggesting the presence of additional complement evasion strategies (204). While *in vitro* studies demonstrate BclA, a FH binding protein of *Bacillus anthracis*, downregulates complement activation and protects against cell lysis, inoculation of mice with a lethal dose of *B. anthracis* spores lacking BclA, did not affect animal survival or bacterial burden compared to inoculation with an isogenic wild type strain (72).

While some studies convincingly demonstrate FH binding is dispensable for pathogen virulence, others have led to inconclusive findings. An example of disputable findings regarding the effect of FH binding in pathogen virulence involves studies of *S. pyogenes*. *S. pyogenes* is a human-specific pathogen that binds human FH through three proteins, including M protein (153). To study the effects of *S. pyogenes* complement evasion *in vivo*, transgenic mice expressing human FH and C4BP were generated. The human FH and C4BP in the serum from these animals, bound to *S. pyogenes in vitro* (205). Compared to wild type animals, transgenic mice expressing FH and C4BP were more susceptible to fatal infection by a *S. pyogenes* strain (AP1 strain) that binds human FH and C4BP through protein H (205), a member of the M protein family (206, 207). This demonstrates the additive virulence effect of binding more than one complement regulator (205). However, infection with a different *S. pyogenes* strain that does not bind FH or C4BP resulted in the same degree of mortality between transgenic and wildtype animals (205). Furthermore, pathogen inoculation with an isogenic *S. pyogenes* strain that does not express protein H, did not affect animal survival (205). Altogether, these results demonstrate recruitment of complement inhibitors by *S. pyogenes* exacerbates infection when FH and C4BP are present, but have no effect on disease if the strain cannot capture the regulatory proteins (205). However, in another study, FH binding to *S. pyogenes* did not modulate pathogen virulence. In this study, transgenic mice expressing chimeric FH (containing human FH CCPs 6–8) did not show an increased susceptibility to infection with a *S. pyogenes* strain capable of binding FH when compared to wild type animals (155). As suggested by Ermert et al., the discrepancy in findings may be due to differences in mouse strains, bacterial strains, and infection route (205).

## Factors Affecting Pathogen Factor H Binding Protein Expression

FH binding proteins are not always constitutively expressed, and instead, expression is governed by numerous regulatory mechanisms described in the following section.

### Environmental Stimuli Influence Factor H Binding Protein Expression

Pathogens are exposed to a wide variety of conditions within the host including transitions between hosts, nutrient supplies, and temperature, all of which have been shown to influence expression of FH binding protein.

FH binding protein expression is associated with nutrient availability within the pathogen environment. For example, expression of the FH binding protein, high-affinity glucose transporter 1 (Hgt1p) in *C. albicans* is highest at low, physiological, glucose concentrations compared to high glucose concentrations (168), which is likely due to the canonical role of Hgt1p in glucose metabolism (208). In *Streptococcus agalactiae*, zinc availability influences expression of FH binding proteins, Sht and ShtII (141). Purified Sht and ShtII proteins bind FH, suggesting a potential role in complement evasion. However, when bacteria survival was assessed in whole blood assays, no protective effect from Sht family proteins was observed. Though Sht and ShtII can bind FH, the primary role of these proteins is bacterial zinc acquisition. Thus, under conditions of low bioavailable zinc, Sht and ShtII expression is activated. In serum, adequate zinc levels repress Sht family gene expression, thus FH binding proteins may only participate in complement evasion under conditions of low zinc.

In some instances, FH binding protein expression is temperature dependent. In *N. meningitidis*, FHbp functions as a thermosensor when its expression increases with temperature (209, 210). *N. meningitidis* resides as harmless flora in the upper airway and enters the bloodstream during invasive disease where it encounters the complement system. Bloodstream temperature is warmer than that of the upper airway which, as suggested by Loh et al., may explain the upregulation of FHbp expression under conditions of increased temperature (210). Likewise, according to Kraiczy et al., temperature dependent upregulation of CRASPs occurs in cultured *Borrelia* (77), which may reflect differential regulation of CRASPs between mammals (higher temperatures) and ticks (lower temperatures) (77).

Similarly, binding outcomes of FH differ between *N. meningitidis* and *N. gonorrhoeae* to accommodate their respective environmental niches [reviewed in (211)]. *N. meningitidis* is an encapsulated organism, which enables high-level complement evasion (212). In contrast, *N. gonorrhoeae*, residing in the genitourinary tract, is not capsulated, and thus relies on host complement regulators for serum resistance. *N. gonorrhoeae* limits complement activity on its surface more effectively *via* FH binding through PorB.1B compared to FHbp of *N. meningitidis* (123). As noted by Shaughnessy et al., the functional differences in binding FH may, in part, be explained by the distinct niches in which these organisms inhabit. FH is very abundant in blood and therefore, likely easily recruited by *N. meningitidis*. In comparison, FH is present in low levels in the genitourinary tract, demanding efficient recruitment by *N. gonorrhoeae* (123).

### Pathogen Life Cycle Affects Factor H Binding Protein Expression

Some pathogens reside in numerous hosts as part of a life cycle and must adapt accordingly to the changing host environment by modulating gene expression, including regulation of FH binding proteins. For example, *Borrelia* participate in an enzootic life cycle between *Ixodes* tick vectors, and mammal, bird, and reptile hosts [reviewed in (213)]. Interestingly, expression of CRASPs reflects the needs of the spirochete in response to its changing environment. CspA (CRASP-1) is produced by *Borrelia* during tick-to-mammal and mammal-to-tick transmission, but is not expressed during established infection (214) or when *Borrelia* is cultured in environments mimicking host conditions (215). In contrast, CspZ (CRASP-2) expression increases in *Borrelia* at the tick bite site in the mammal and is maintained throughout infection, but is undetectable in the tick (214). CspZ gene and protein expression is also induced when *Borrelia* are treated with human blood and its expression is required for *Borrelia* bacteremia and tissue colonization in mice (216). In this particular study, even though mice were infected using spirochetes previously subjected to human blood, which does not reflect the natural life cycle of *Borrelia*, it nevertheless exemplifies the influence of human blood on *Borrelia* gene expression (217).

In another example, *Borrelia turicatae* mRNA expression of the gene for the FH binding protein, FhbA, was lower in *Borrelia* from fed ticks compared to *in vitro* culture; however the functional role of FhbA in infection remains to be determined (218). Finally, OspE paralogs (CRASPs 3–5) are expressed during all stages of mammalian infection [reviewed in (83, 219)]. Concurrent expression of CRASPs during the spirochete infection cycle confounds interpretation of the role each protein plays in complement evasion [reviewed in (83)]. Instead of contributing separately to complement evasion, CRASPs may work synergistically to carry out their functions as suggested by Bykowski et al. (214).

Some pathogens including protozoans differentiate into unique morphological life stages and FH binding capabilities vary concordantly. *P. falciparum*, a protozoan parasite, recruits FH using unique FH binding proteins according to the parasite life stage (172, 173). The invasive blood-stage merozoite found in humans uses Pf92 to bind FH (173), while extracellular gametes residing in the mosquito midgut, recruit human FH from a blood meal using a different FH binding protein, PfGAP50 (172). In *Trypanosoma brucei*, the proliferating slender form expresses the lowest levels of FH binding protein, FH receptor (FHR), followed by the quiescent stumpy form, and finally, the procyclic form expresses the highest levels of FHR (169). Both the slender and stumpy forms are present in mammalian blood, whereas the procyclic form is present in the midgut of the tsetse fly vector [reviewed in (220)]. In a related example, the FH binding proteins of *C. albicans*, Pra1, is upregulated upon switching from yeast to hyphal growth, which may explain, in part, why the hyphal form is more invasive than the yeast (164).

### Pathogen Strain and Passage Number Affects Factor H Binding Protein Expression

Expression of FH binding proteins is subject to variability amongst pathogen strains. For example, PspC expressed by *S. pneumoniae* undergoes sequence variation leading to changes in FH binding capacity (221). Antibodies raised against a particular PspC variant successfully prevented FH binding and *in vivo* opsonophagocytic bacterial killing, but did not cross-react with *S. pneumoniae* that contained different sequence variations of PspC (221). Similarly, in *S. pneumoniae*, serotype invasiveness correlates with FH binding (222). Additionally, *S. pyogenes* bind FH through expression of M proteins. M proteins exhibit sequence variation between strains, and hence, vary in the ability to bind FH (155). Furthermore, analysis of FHbp expression across a panel of serogroup B meningococcal strains revealed the level of FHbp expression varies by at least 15-fold, and that variant 1, which is a component of meningococcus vaccines (discussed later), expresses significantly more protein than variant 2 or 3 strains (223). Expression of FH binding proteins Pra1 and Gpm1 is conserved across clinical isolates of *C. albicans*; however, expression levels vary, and higher expression levels correlate with FH binding (224). Moreover, FH binding varies from 5-42% across patient strains of *Fusobacterium necrophorum*, and strains which bound FH strongly result in more severe infections (101).

Culture passage number also influences FH binding expression. *Leptospira* strains with few passages display higher FH binding expression compared to culture attenuated strains, suggesting pathogens lose the ability to bind FH in culture (225). Similarly, passage number also decreases expression of CRASP-1, -2, and -5 in *Borrelia afzelii* (77). Passage number is postulated to suppress CRASPs expression in *B. garinii* because isolates from patients with neuroborreliosis bind FH, whereas strains with prolonged growth *in vitro*, do not (226).

### Localization of Factor H Binding Proteins

FH binding proteins assume strategic spatial positioning to promote efficient evasion of the AP. This is evidenced by PspC of *S. pneumoniae*. Pneumococci divisional septa are insufficiently protected by the bacterial capsule, especially at the site where cell separation is initiated, permitting entry of serum factors and allowing complement activity (227). PspC compensates for the breach of protection by localizing to the cell septa to control lateral complement amplification (227).

While most FH binding proteins are surface bound, some are secreted. For example, Pra1 of *C. albicans* is secreted and regulates FH in the fluid phase (164). Pra1 is also surface expressed and localizes primarily at the tip of cells, which suggests an important role of Pra1 upon contact with host tissues and surfaces during infection (164). Likewise, Hgt1p of *C. albicans* is present on the cell wall, cell membrane, and intracellular and extracellular vesicles (168).

## Therapeutic and Preventive Strategies Targeting the Pathogen-Factor H Interface

**Figure 4:**
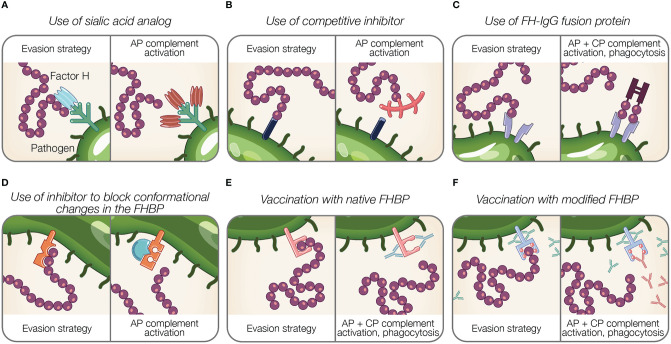
Potential therapeutic and preventive strategies targeting the interaction between Factor H and pathogens. **(A–F)** Theoretical representations of pathogen evasion strategies targeted for therapy and prevention are shown. For each box, the left panel illustrates a pathogen evasion strategy and the right panel represents the intended result of therapeutic intervention, which includes AP and CP activation, and phagocytosis. **(A)** Sialic acid species (shown in light blue) are replaced by analogs unable to recognize FH (shown in brown). **(B)** Competitive inhibitors for FH binding proteins (FHBP) prevent FH interaction with the pathogen surface. **(C)** FH fusion proteins containing the Fc portion of IgG fused to FH binding fragments (CCPs 6–7, 19–20) bind FHBP to activate the CP *via* antibody recognition, facilitate Fcγ receptor-mediated phagocytosis, and prevent FH binding to the pathogen surface. **(D)** Inhibitors prevent conformational change of FHBP required to enhance their binding to FH. **(E)** Vaccination with native FHBP. **(F)** Vaccination with modified FHBP unable to bind FH, reveals FHBP epitopes (red balls) to improve immunogenicity.

Acquiring host complement negative regulators, such as FH, allow pathogens to bypass complement attack and persist in the host. Hence, strategies directed against the FH-pathogen interface hold therapeutic value. The following section describes existing and potential approaches to target pathogen binding of FH (**Figure 4**).

### Interference of Pathogen Sialic Acid Expression

In addition to the role of sialylation in pathogen FH acquisition, it also modulates other pathways of complement, as well as phagocytosis and epithelial invasion [reviewed in (189)]. The importance of sialylation in pathogenesis renders it an enticing therapeutic target (**Figure 4A**). In the case of gonococci that are becoming increasingly antibiotic resistant, this approach is timely and reviewed elsewhere (188, 189, 228).

Gonococci incorporate sialic acid in surface glycans (LOS) resulting in FH binding [reviewed in (189)]. A strategy that manipulates pathogen sialylation to disable complement evasion, involves supplying gonococci with sialic acid analogs that do not bind FH. When such analogs are incorporated into pathogen glycans, it renders bacteria susceptible to complement and substantially reduces bacterial survival in normal human serum (196, 229). Intravaginal administration of a sialic acid analog (CMP-Leg5,7Ac_2_) to transgenic mice capable of expressing sialic acid compounds found in humans protects against multidrug-resistant gonococci (229). Moreover, this sialic acid analog was stable at vaginal pH and temperature and did not incorporate into glycans on host surfaces, which is noteworthy because incorporation into host glycans may cause an immunogenic response against host tissue (229). The stability and limited side effects of sialic acid analog indicate an effective treatment option (229).

### Inhibitors to Functionally Disable Factor H Binding Proteins or Its Interaction With Factor H

Inhibitors that block the interaction between FH and FH binding proteins can be considered as potential therapeutics (**Figures 4B, D**). As described previously, FHbp of *N. meningitidis* binds FH using a site shared by SOS, a highly sulphated analog of GAGs (122). The addition of SOS inhibits FH interaction with FHbp *in vitro*, thus as suggested by Schneider et al., provides support for small molecule competitive inhibitors in disabling pathogen evasion (122). However, competitive inhibitors binding FH may also interfere with host regulation of complement activity.

The binding of FH to SdrE of *S. aureus* is enhanced after a conformational change in the binding protein from a resting, locked state, to an open state (136). Small molecules inhibitors may have a potential in preventing conformational changes of FH binding proteins required to bind FH, similar to what occurs for SdrE [reviewed in (199)].

### Factor H-Fc Fusion Proteins

Targeting the pathogen-FH interface is accomplished using fusion proteins in which FH domains essential for surface recognition (CCPs 18–20 or 6–7) are fused to the Fc region of IgG. Fusion proteins are designated FHX-X/Fc, where X-X refers to the FH domains used in the fusion protein. The proposed mechanisms of fusion proteins are threefold: (a) prevent FH binding to the microbial surface; (b) activate the CP *via* antibody recognition; and (c) facilitate Fcγ receptor-mediated phagocytosis [reviewed in (189)] (**Figure 4C**). Fusion proteins are advantageous because identifying pathogen FH binding proteins or ligands is not required. However, determining FH domains involved in pathogen binding is necessary to design applicable fusion proteins.

Animal model studies demonstrate the efficacy of fusion proteins using FH CCP 6–7. In infant rats, administration of FH6-7/Fc prior to intraperitoneal inoculation of a serogroup C strain of *N*. *meningitidis* dose-dependently reduced bacterial blood burden (230). Similarly, concurrent intranasal administration of FH6-7/Fc with inoculation of non-typeable *H. influenzae* reduced mouse lung bacterial burden (129). In a model of *S. pyogenes* sepsis, intranasal FH6-7/Fc treatment of human FH transgenic mice resulted in decreased animal mortality (231). Additionally, fusion proteins with CCPs 18–20 bound to gonococci and resulted in complement-dependent bactericidal activity (119).

Given the importance of CCP 6–8 (232–236) and CCP 19–20 (237, 238) in discriminating self from non-self surfaces, fusion proteins containing these domains may outcompete full-length FH for binding host cells, resulting in unwanted complement-mediated attack. In order for fusion proteins utilizing CCP 19–20 to be effective therapeutic solutions, these domains must be modified to prevent host cell interaction. This was accomplished by the generation of a CCP 19–20 fusion protein containing a point mutation in CCP 19 (D1119G). This mutation results in lower affinity for C3b binding compared to wild type CCP 19–20 restricting its ability to bind human cells, even while maintaining normal binding affinity for polyanions (238). D1119G bound to highly sialylated gonococci in the presence of serum, resulting in a robust *in vitro* complement response (239). Moreover, therapeutic administration of D1119G reduced infection duration and burden in a mouse vaginal colonization model of *N. gonorrhoeae* without affecting lysis of human erythrocytes (239). Similarly, FH CCP 19–20 fragments containing the D1119G mutation also bound to whole *P. aeruginosa*, *H*. *influenzae*, *Bordetella pertussis*, *S*. *pneumoniae*, and *C*. *albicans* organisms (74), suggesting the therapeutic relevance of mutant FH fusion proteins against numerous pathogens.

### Factor H Binding Proteins as Vaccines

A suitable vaccine candidate is (a) immunogenic; (b) conserved across different strains and genospecies of the respective pathogen; (c) expressed during human infection; (d) necessary for development of a clinical infection; (e) surface exposed; and (f) raises an immune response that neutralizes an important virulence determinant as proposed by Bhattacharjee et al. and Meri et al. (85, 240). FH binding proteins meet many of these requirements, and thus are viable targets for vaccine development. This section describes clinically approved (**Figure 4E**) and emerging vaccines (**Figure 4F**) utilizing FH binding proteins.

#### Clinically Approved Vaccines Utilizing Factor H Binding Proteins

FHbp is a successful vaccine target against *N. meningitidis*, the causative agent of invasive meningococcal disease. Five of the six serogroups of *N*. *meningitidis* express capsular polysaccharides that are effective vaccine targets. However, because the capsule of serogroup B is poorly immunogenic, alternative vaccine targets are necessary to protect against the high mortality and morbidity associated with meningococcus disease [reviewed in (241)]. FHbp is a viable therapeutic target because it is widely expressed across numerous meningococcus B isolates [reviewed in (241)]. Two licensed vaccines, 4CMenB and MenB-FHbp, both containing FHbp, are approved to protect 10–25 year old individuals against serogroup B meningococcus [reviewed in (241, 242)].

FHbp displays sequence variability with 3 main variants belonging to subfamilies A (variant 2 and variant 3) and B (variant 1) (243, 244). Consequentially, immunization with vaccines representing single variants do not offer protection against strains containing other variants (243–245). MenB-FHbp is a bivalent vaccine containing recombinant lipidated FHbp from subfamily A variant A05 and subfamily B variant B01 [reviewed in (242)]. Because FHbp from two variants are represented in this vaccine, MenB-FHbp offers broad coverage against multiple *N. meningitidis* FHbp variants (246). In contrast, 4CMenB is a multi-component vaccine where one of the components is a FHbp that represents only one variant (subfamily B) [reviewed in (247)].

Sequence variability limits the degree of protection against FHbp variants. For example, post immunization sera from mice injected with a single variant did not offer *in vitro* cross protection against other variants (245). Thus, sequence variability of FHbp poses difficulties in vaccine development. However, recent studies have introduced alternative vaccine candidates that elicit antibody responses against FHbp variants 1, 2, and 3 to offer broad protection against meningococcal disease. One such promising candidate is the Gonococcal homologue of meningococcal FHbp (Ghfp) that protects against *N. meningitidis* expressing any of the 3 FHbp variants (248). Vaccination with chimeric FHbp antigens is an additional approach for offering broad protection. Scarselli et al. developed a chimeric FHbp antigen in which the surface is modified to confer specificity against all three variants, resulting in cross protection in mice (249). Another example includes chimeric vaccines developed by fusing domains conserved across all FHbp variants and selective portions of domains from variant 1 FHbp and domains of variant 2 FHbp (250). These molecules were found to elicit bactericidal activity against strains expressing the different variants (250). Chimeric antigens can also be generated by fusing FHbp with other immunogenic antigens such as the VR2 epitope from the integral membrane protein PorA that induces an immunogenic response against *N. meningitidis* in mice (251).

Though approved for clinical use, understanding the scope of FHbp-based antibodies remains an area of active research. One such area involves long-term risks of vaccination, such as the development of anti-FH autoantibodies. Interaction of FHbp (delivered as a vaccine) with FH may result in the development of autoantibodies against FH. A few studies have investigated this potential outcome. Sharkey and colleagues noted that 2.5% of individuals vaccinated with 4CMenB show an increase in anti-FH autoantibodies (a low level of anti-FH autoantibodies are present in some individuals (252)). However, this increase was transient, and no adverse effects were reported in individuals with higher anti-FH autoantibodies (253). In another study, 4CMenB immunization resulted in the development of anti-FH autoantibodies in Rhesus macaques (254). Since the long-term effects of FH autoantibodies are still not clear, further investigation is required to assess the risk of autoimmune disease in response to meningococcal vaccines, as suggested by Sharkey et al. (253).

Other avenues of research into FHbp vaccines include expanding applications and improving efficacy. Recently, antibodies raised in response to MenB-FHbp were shown to be effective against non-serogroup B meningococci in serum bactericidal assays (255), suggesting this vaccine may provide broad protection against meningococcal disease. Additionally, vaccine delivery with polyhydroxybutyrate beads engineered to display FHbp antigens, show promise in preclinical studies as means to overcome limitations of recombinant protein vaccines such as poor immunogenicity and adjuvant requirements (256). Finally, recent studies using vaccines containing mutant FHbp with less ability to bind FH combined with native outer membrane vesicles showed higher serum bactericidal activity than 4CMenB vaccination (254). This vaccination approach also generated less FH autoantibodies than 4CMenB (254).

#### Emerging Vaccines Utilizing Factor H Binding Proteins

##### Vaccination With Native Factor H Binding Proteins

While vaccines against *N. meningitidis* are the only clinically approved therapies targeting the pathogen FH interface, other examples are emerging. The FH binding protein, PspC of *S. pneumoniae*, is a promising vaccine target. Mice immunized with a PspC fragment containing the FH binding domain conferred protection when challenged with the same strain used for immunization (257). Antibodies from immunized animals enhanced CP activity, but also competed for human FH binding, suggesting interference with the AP (257). However, antibodies generated against PspC from immunized human sera do not recognize the FH binding site, suggesting FH masks epitope recognition (258).

Vaccination with FH binding proteins is also effective against *Leptospira interrogans*. Immunization with a multi-subunit, adjuvant vaccine comprising multiple FH binding proteins from *L. interrogans* had similar protective efficacy and survival rate in hamsters challenged with *L. interrogans* compared to monovalent vaccine administration containing only LigAc (259). Though results indicate LigAc is likely a superior vaccine antigen in the multi-subunit vaccine, only the multi-subunit vaccine reduced leptospiral renal colonization in surviving animals (259). Further investigation is needed to identify the FH binding proteins in this observed effect from multi-subunit vaccination (259).

##### Vaccination With Modified Factor H Binding Proteins

An innovative approach towards applying FH binding proteins for vaccination involves manipulating FH binding proteins. Modification of FH binding proteins from several organisms has been shown to disable FH binding (**Figure 4F**).

Murine immunization with a non-binding FH mutant of *N. meningitidis* FHbp resulted in antibodies with a higher bactericidal activity than native FHbp vaccination (260). This finding suggests binding of FH to the native FHbp vaccine can decrease a protective antibody response and that mutant FH binding proteins may serve as a superior vaccine (260). Importantly, these studies utilized transgenic mice expressing human FH because FHbp only binds human FH (261).

As discussed above, PspC vaccination fails to generate antibodies against the FH binding site (258). Thus, as suggested by Glennie et al., generation of a modified PspC unable to bind FH presents a viable vaccine opportunity given the role of this protein in complement evasion and host cell invasion (258).

A recent study in *Borrelia* eloquently demonstrates the ability of mutated FH binding proteins to confer protective immunization. CspZ (CRASP-2) belongs to a family of lipoproteins utilized by *B. burgdorferi*, a causative agent of Lyme disease, to evade complement. CspZ binds FH (77) though the degree of binding varies across an extensive panel of human Lyme disease isolates (262). CspZ is an enticing therapeutic target because it is immunogenic in humans (though titers vary between individuals (262)) and its expression is highly conserved among species associated with Lyme disease [reviewed in (219)]. However, CspZ on its own is not protective against *B. burgdorferi* infection. Immunization with recombinant CspZ did not impact *B. burgdorferi* infection (262, 263) or prevent Lyme disease pathology in a mouse model (263).

Nonetheless, studies by Marcinkiewicz and colleagues revealed the potential of CspZ as a therapeutic target by using a nonbinding mutant of CspZ (CspZ-YA). Because CspZ-YA cannot bind FH, new epitopes previously cloaked by FH binding are revealed (216). As a result, the immunogenicity of CspZ increases and prevents *Borrelia* colonization (216). CspZ-YA conjugated to virus-like particles (VLP-CspZ-YA) protects passively immunized mice from Lyme infection (216). Additional work in mice infected by *Ixodes scapularis* nymphal ticks carrying *Borrelia* demonstrated VLP-CspZ-YA protects against spirochete tissue colonization and arthritis development (264). These authors suggest anti-CspZ-YA antibodies bind CspZ at epitopes within the FH binding site, thus competing with FH binding (264). Furthermore, CspZ-YA vaccination protected mice against a strain of *B. burgdorferi* that does not bind FH and immunized sera eliminated this strain *in vitro*, suggesting resulting antibodies may target CspZ through the CP (264).

## Potential Targeting of Factor H in Other Infectious Diseases

While FH interactions among bacteria are well-described and applied in clinical therapy, further research in this field is warranted for other classes of pathogens including protozoa, helminths, viruses, and fungi. Closing this gap is relevant for developing treatments for diseases caused by pathogens known to evade complement. The following sections provides preliminary support for pathogens that interact with FH, but require further work to harness these findings for therapy.

### Factor H Interactions With Protozoa

The protozoan, *Trypanosoma cruzi* is the causative agent of Chagas disease, which progresses from an acute, often undiagnosed, asymptomatic stage, to a deadly chronic stage of which 30–40% of chronically infected individuals develop cardiomyopathy, mega-syndromes, etc. [reviewed in (265)]. Currently, there are no vaccines for *T. cruzi* nor treatments available for the chronic, fatal stage of the disease. *T. cruzi* adopts various strategies to evade complement, including the AP [reviewed in (266)]. When the infective form of *T. cruzi* (trypomastigotes), which is normally resistant to complement-mediated killing, is treated with enzymes including sialidase and trypsin, the parasites become susceptible to complement-mediated lysis in human serum (267), suggesting parasites may be protected from the AP by FH that has been hijacked by surface-bound sialic acid. This notion is also supported by data showing trypomastigotes that are pre-opsonized with C3b bind FH (170). However, the molecular mechanisms involved in binding remain unknown. Likewise, as suggested by Sikorski et al., the mechanism by which *Toxoplasma gondii* captures FH may be through expression of sialic acid or heparan sulfated proteoglycans (174). In addition, *P. falciparum*, the causative agent of malaria, has developed resistance to all antimalarial drugs used against it [reviewed in (268)]. FH has been shown to bind *P. falciparum*, though not all receptors have been identified (171) and doing so holds promise for therapeutic avenues against drug resistance. Overall, interfering with the ability of infectious protozoa to bind FH represents a possible treatment strategy against diseases caused by these pathogens; however, more work is required to understand the molecular mechanisms involved in these interactions with FH.

### Factor H Interactions With Helminths

*Echinococcus granulosus*, a zoonotic cestode, causes cystic echinococcosis in humans when larvae develop cysts in organs including liver, lungs, brain, and bones [reviewed in (269)]. FH binds *E. granulosus* through interactions involving the neutral, sugar-rich, laminated layer of the cyst wall (176). Myoinositol hexakisphosphate (InsP_6_) is a protein abundantly expressed in the laminated layer and is shown to bind FH through *in vitro* studies with purified protein (270). However, studies representing physiological conditions suggest InsP_6_ does not protect against AP-mediated attack and may instead, activate the CP through C1q recognition (270). Thus, the mechanism for FH interaction with *E. granulosus* remains largely unresolved and likely involves other components on the laminated layer other than InsP_6_ [reviewed in (271)].

*Onchocerca volvulus* is a parasitic nematode causing onchocerciasis, or river blindness. Novel treatment options are necessary for this disease given the threat of drug resistance to the standard therapy, ivermectin [reviewed in (272)]. The pathogenic stage of *O. volvulus* (microfilariae) bind FH using CCPs 8–20 with preservation of cofactor activity, but the binding ligand remains unknown (175). *Loa Loa* is another parasitic nematode and the causative agent of loiasis, characterized by subconjunctival eye passage of the adult worm, angioedema, and pathology associated with the lungs, brain, heart, and kidneys [reviewed in (273)]. Patients with a high infectious load are at risk for severe complications from ivermectin treatment (274), thus alternative treatment options are important. Larval *L. Loa* acquires FH on the outermost sheath layer and retains its cofactor activity (177). However, FH CCPs involved in this reaction are not yet described, but likely does not involve heparin binding domains (177).

### Factor H Interactions With Viruses

There are many examples of viruses evading complement [reviewed in (62)], but few associate complement evasion to pathogen interactions with FH. West Nile virus binds FH using non-structural protein (NS1); however, the mechanisms of this interaction is unknown (178). Interestingly, serum levels of NS1 correlate with disease severity and viremia [reviewed in (275)], which may be due in part, to the ability of NS1 to capture FH.

Human immunodeficiency virus-1 (HIV-1) is resistant to complement lysis in circulation even though HIV-1 and anti-HIV-1 antibodies activate complement [reviewed in (276)]. FH binds the HIV-1 envelope proteins gp41 and gp120 (179–182). More robust studies are required to understand the role of FH binding in HIV-1 complement evasion.

## Conclusion

Numerous pathogens exploit host FH, the primary negative regulator of the AP of complement, as part of a robust complement evasion strategy. Characterizing interactions between pathogens and FH provides insight into the failure of FH to discriminate self from non-self, and/or the impressive resourcefulness of pathogens to outsmart complement regulation. The biological relevance of binding FH is demonstrated by established and emerging vaccines targeting the pathogen-FH interface and evidenced by the number of pathogens utilizing FH and expressing multiple FH binding proteins. Additionally, studying pathogen interactions with FH may reveal more information about how FH operates in the host. The selective pressure of pathogens to maintain FH binding capabilities suggest undiscovered details about the role of complement in immune defense against pathogens.

## Author Contributions

SRM, SSM, and VF conceived and wrote the paper. CC critically reviewed and edited the paper. SRM, SSM, and CC designed the figures. All authors contributed to the article and approved the submitted version.

## Funding

This work was supported by National Institute of Health R01HL112937 (VF), The University of Toledo Biomedical Research Innovation Program (VF), and The American Association of Immunologists Careers in Immunology Fellowship Program (VF and SSM).

## Conflict of Interest

VF serves as a consultant for, and receives grant funding from, Apellis Pharmaceuticals.

The remaining authors declare that the research was conducted in the absence of any commercial or financial relationships that could be construed as a potential conflict of interest.
